# Early outcomes after venous thromboembolism in Hispanic and non-Hispanic patients: insights from the Registro Informatizado de Enfermedad TromboEmbólica Registry

**DOI:** 10.1016/j.rpth.2026.106969

**Published:** 2026-09-15

**Authors:** Lizbeth Cabrera, Alfonso Tafur, Leticia Guirado-Torrecillas, Jose Maria Pedrajas, Fares Moustafa, Concepción Falgá, Aída Gil-Díaz, Andris Skride, Javier Pagán Escribano, Manuel Monreal, Registro Informatizado de Enfermedad TromboEmbólica Investigators, Manuel Monreal, Paolo Prandoni, Benjamin Brenner, Dominique Farge-Bancel, Raquel Barba, Peter Verhamme, Hugo Hyung Bok Yoo, Radovan Malý, Laurent Bertoletti, Sebastian Schellong, Inna Tzoran, Pierpaolo Di Micco, Abilio Reis, Marijan Bosevski, Lucia Mazzolai, Joseph A. Caprini, My Hanh Bui, A. Abad-Fernández, L. Afonso-Nuez, P. Agudo, J. Aibar, A. Alberich-Conesa, J. Álvarez-Albarrán, C. Amado, M. Angelina-García, J.I. Arcelus, A. Ballaz, R. Barba, M. Barrón, B. Barrón-Andrés, J. Bascuñana, F. Beddar-Chaib, Á. Blanco-Molina, I. Cabello-Rojano, N. Chamorro, L. Chasco, G. Claver, J. Creu-Paris, Criado J, Juana-Izquierdo C. De, J. Del Toro, P. Demelo-Rodríguez, J.D. Díaz-Anichiarico, M.C. Díaz-Pedroche, J.A. Díaz-Peromingo, D. Durán, P. Esquerdo-Serrano, C. Falgá, C. Fernández-Martínez de Septién, J. Ferreiro-Celeiro, Á. Fidalgo, A. Formica, I. Francisco, C. Gabara, F. Galeano-Valle, F. García-Bragado, A. García-Ortega, O. Gavín-Sebastián, A. Gil-Díaz, E. Girona, C. Gómez-Cuervo, J. González-Olmedo, I. Gorostidi-Álvarez, J. Gorostidi-Pérez, L. Guirado, J. Gutiérrez-Guisado, L. Hernández-Blasco, J. Hernández-Borge, D. Jiménez, R. Jiménez, I. Jou, M.D. Joya, A. Larrauri, P. Llamas, P. López-Miguel, J.J. López-Núñez, A. López-Ruiz, J.B. López-Sáez, A. Lorenzo, P. Lucas-García, O. Madridano, P.J. Marchena, M. Martín-del Pozo, M. Monreal, L. Monzón-Escribano, A. Moreno-Fernández, J.A. Nieto, M.J. Núñez-Fernández, M. Olid, L. Ordieres-Ortega, M. Ortiz, S. Otálora, J. Pagán, P. Parra-Caballero, J.M. Pedrajas, M. Pérez-Pinar, C. Pérez-Ductor, M.L. Peris, M.L. Pesce, G. Puche, A. Rodríguez-Cobo, M.D. Rojas-Piñero, P. Ruiz-Artacho, N. Ruiz-Giménez, M. Sánchez-Camacho, T. Sancho, V. Sendín, T. Sidawi-Urbano, G. Sierra-Palomares, P. Sigüenza, L. Sindín-Martín, A. Solé, L. Teres-Pueyo, M.R. Toda, J. Trujillo-Santos, L. Tudela, R. Valle, J.F. Varona, E. Vega-Romero, P. Villares, C. Ay, S. Nopp, I. Pabinger, M. Erard, Q. Van Thillo, P. Verhamme, H.H.B. Yoo, S.E. Carbonell, J.E. Gómez-Mesa, A.C. Montenegro, J. Roa, P. Grenar, J. Hirmerova, R. Malý, S. Accassat, S. Benarroch, L. Bertoletti, A. Bura-Riviere, J. Catella, R. Chopard, R. Duong, O. Espitia, B. Leclercq, I. Mahé, G. Millet, F. Moustafa, G. Poenou, G. Sarlon-Bartoli, S. Schellong, Y. Jenab, A. Khodayari, P. Sadeghipour, S. Yadangi, B. Brenner, N. Dally, I. Tzoran, G. Barillari, F. Bilora, C. Bortoluzzi, M. Bortoluzzi, B. Brandolin, G. Buso, M.M. Ciammaichella, P. Di Micco, A. Guida, E. Imbalzano, A. Licci, C. Marcon, D. Mastroiacovo, U. Mazzarelli, R. Pesavento, V. Pizzuti, A. Poz, P. Prandoni, M. Provisione, P. Scarinzi, A. Visonà, N. Vo Hong, B. Zalunardo, H. Cesnieks, A. Skride, M. Zicans, S. Fonseca, M.D. Martins, C. Pereira-Fontes, M. Bosevski, M. Boshevska, S. Barco, L. Mazzolai, D.J. Angiolillo, B. Bikdeli, J.A. Caprini, L. Ortega-Paz, A.J. Tafur, M.H. Bui, S.T. Nguyen, G.B. Tran

**Affiliations:** 1 University of Chicago, Pritzker School of Medicine, Chicago, Illinois, USA; 2 Endeavor Health, Vascular Medicine, Evanston, Illinois, USA; 3 Department of Internal Medicine, Hospital Universitario Virgen de la Arrixaca, Murcia, Spain; 4 Department of Internal Medicine, Hospital Clínico San Carlos, Madrid, Spain; 5 Emergency Department, University Hospital of Clermont-Ferrnad, Clermont-Ferrand, France; 6 Hospital de Mataro, Barcelona, Spain; 7 Servicio de Medicina Interna, Hospital Universitario de Gran Canaria Doctor Negrín, Las Palmas, Spain; 8 Department of Internal Medicine, Riga Stradiņš University, Latvia; 9 Department of Internal Medicine, Morales Meseguer General University Hospital, Murcia, Spain; 10 Faculty of Health Sciences, UCAM - Universidad Católica San Antonio de Murcia, CIBER Enfermedades Respiratorias (CIBERES), Madrid, Spain

**Keywords:** anticoagulation, ethnicity, health disparities, Hispanic or Latino, major bleeding, outcomes assessment, pulmonary embolism, venous thromboembolism

## Abstract

**Background:**

Racial and ethnic differences in venous thromboembolism (VTE) incidence have been reported, but Hispanic patients remain underrepresented in VTE cohorts, and their 90-day postdiagnosis outcomes remain incompletely characterized.

**Objectives:**

To compare 90-day risks of recurrent VTE, major bleeding, and mortality between Hispanic and non-Hispanic patients with acute VTE.

**Methods:**

Exposure-matched analysis of the Registro Informatizado de Enfermedad TromboEmbólica Investigators registry, a prospective, international cohort of adults with acute VTE. Hispanic patients were matched 1:3 to non-Hispanic patients by age, sex, index VTE phenotype, active cancer, and calendar period. The matched cohort included 1792 Hispanic and 5376 non-Hispanic patients. Exposure was self-identified Hispanic ethnicity. Main outcomes and measures were 90-day recurrent VTE, major bleeding, and all-cause mortality. Odds ratios (ORs) with 95% CIs were estimated using hierarchical logistic regression.

**Results:**

The matched cohort had a mean age of 57 ± 18 years, and 59% were women. At 90 days, recurrent VTE occurred in 1.6% of Hispanic patients and 1.2% of non-Hispanic patients (absolute difference, 0.4 percentage points); major bleeding occurred in 2.1% and 1.7%, respectively (absolute difference, 0.4 percentage points); the composite outcome occurred in 3.4% and 2.9%, respectively (absolute difference, 0.5 percentage points); and mortality occurred in 4.1% and 3.9%, respectively (absolute difference, 0.2 percentage points). In fully adjusted models, Hispanic ethnicity was associated with increased odds of recurrent VTE (OR, 1.90; 95% CI, 1.10-3.28), major bleeding (OR, 1.73; 95% CI, 1.09-2.75), and the composite outcome (OR, 1.77; 95% CI, 1.23-2.54), but not mortality (OR, 1.05; 95% CI, 0.68-1.64).

**Conclusion:**

In this matched registry analysis, self-identified Hispanic ethnicity was associated with modestly higher adjusted odds of recurrent VTE and major bleeding, but not mortality. Although adjusted relative estimates differed, absolute differences were small. These observational associations should not be interpreted as causal because residual confounding cannot be excluded.

## 1 Introduction

The first 90 days after venous thromboembolism (VTE) represent a period of heightened clinical vulnerability, during which the risks of recurrent thrombosis and anticoagulant-related bleeding are greatest [1,2]. Clinical decisions made during this early phase (including anticoagulant selection, dosing, and follow-up) may substantially influence outcomes [[3], [4], [5], [6]].

Although racial and ethnic differences in VTE incidence and outcomes have been reported, evidence regarding differences in early outcomes after VTE is more limited, particularly among Hispanic patients [[7], [8], [9], [10]]. Recent epidemiologic data from Latin America have also described variation in VTE presentation, management, and outcomes across health care systems [11,12]. However, it remains uncertain whether 90-day risks of VTE, major bleeding, and mortality differ between Hispanic and non-Hispanic patients after an acute VTE. Examining this early treatment period is clinically relevant because decisions regarding anticoagulant selection, dosing, monitoring, and transitions of care occur while patients remain at increased risk for both recurrence and bleeding [13,14]. This represents a distinct knowledge gap that is not resolved by existing incidence data or by underrepresentation alone.

Hispanic ethnicity is a broad, self-identified sociocultural classification encompassing heterogeneous ancestral, cultural, geographic, and socioeconomic backgrounds rather than a discrete biological exposure [15,16]. Associations between ethnicity and clinical outcomes may reflect multiple measured and unmeasured factors, including patient characteristics, access to care, treatment patterns, and social determinants of health. The present analysis was not designed to distinguish among these potential explanations.

We evaluated early outcomes after acute VTE among Hispanic and non-Hispanic patients in the Registro Informatizado de Enfermedad TromboEmbólica Investigators (RIETE) registry using a prespecified exposure-matched design. Our objective was to compare 90-day risks of recurrent VTE, major bleeding, and mortality.

## Methods

### Study design and data source

We performed an exposure-matched cohort analysis using data from the RIETE (Registro Informatizado de Enfermedad Tromboembólica) registry, an ongoing, multicenter, prospective international registry that enrolls consecutive adult patients with objectively confirmed acute VTE, including deep vein thrombosis (DVT) and pulmonary embolism (PE). The registry collects standardized information on patient characteristics, treatment strategies, and clinical outcomes during routine clinical care [17].

All participants obtained local ethics approval, and all patients provided informed consent according to local regulatory requirements.

### Study population

We included adults aged ≥18 years with acute symptomatic lower-limb DVT and/or PE who were enrolled after systematic collection of race and ethnicity data was implemented and who had complete information on ethnicity. Patients with missing or ambiguous ethnicity data were excluded.

### Exposure definition

The primary exposure was Hispanic ethnicity, defined by self-identification as Hispanic or Latino at the time of index VTE diagnosis. Hispanic ethnicity represents a heterogeneous population with diverse cultural, geographic, and socioeconomic backgrounds that are not captured in detail within the registry. Accordingly, this variable was interpreted as a broad sociocultural classification rather than a discrete biological category. The comparison group consisted of patients self-identifying as non-Hispanic (including non-Hispanic White, Black, Asian, or Arab). Race and ethnicity were used exclusively to define exposure groups and were not included as matching variables.

### Matching strategy

To reduce confounding by baseline clinical characteristics, we conducted a prespecified exposure-matched analysis. Each Hispanic patient was matched with up to 3 non-Hispanic patients using random sampling within strata defined by age (10-year bands), sex, index VTE phenotype (PE vs isolated DVT), active cancer at baseline (yes/no), and calendar period of diagnosis (5-year bands). When more than 3 eligible comparators were available within a stratum, 3 were selected at random. Geographic region was not included in the matching algorithm. Instead, it was included as a covariate in multivariable models and examined in stratified analyses because the geographic distribution of Hispanic and non-Hispanic patients differed substantially.

### Covariates

Baseline variables included demographic characteristics (age, sex, and body weight), geographic region of enrollment, and clinical features. Prespecified clinical covariates included: active and metastatic cancer, recent surgery and immobilization, pregnancy or hormone-associated VTE, prior VTE, known thrombophilia, renal function, liver disease, heart failure, coronary artery disease, diabetes mellitus, chronic lung disease, anemia and thrombocytopenia. Index event characteristics included VTE phenotype, proximal vs distal DVT location, and hemodynamic instability at presentation for PE.

### Outcomes

The primary outcomes were assessed from the date of index VTE diagnosis through 90 days of follow-up and included recurrent VTE, major bleeding, and all-cause mortality. Secondary outcomes included VTE-related mortality and bleeding-related mortality, defined as death within 10 days of recurrent VTE or major bleeding event, respectively. Outcomes were reported by participating centers according to standardized RIETE registry definitions.

Patients were followed for at least 90 days after VTE diagnosis through routine clinical care. Anticoagulation management and follow-up strategies were determined by treating physicians without protocol-mandated standardization.

### Statistical analysis

Baseline characteristics were summarized as means with standard deviations or medians with interquartile ranges for continuous variables and as counts with percentages for categorical variables. Between-group differences were assessed using standardized mean differences, with values >0.10 indicating meaningful imbalance.

Primary analyses were conducted using logistic regression models to estimate odds ratios (ORs) and 95% CIs for the association between Hispanic ethnicity and 90-day outcomes. Models were specified sequentially: 1) unadjusted models, including ethnicity with stratification by matched sets; 2) clinically adjusted models, including active cancer, provoking factors (recent surgery, immobilization, pregnancy, and hormone-associated VTE), prior VTE, renal insufficiency, defined as creatinine clearance <60 mL/min, liver disease, heart failure, chronic lung disease, thrombocytopenia, anemia, geographic region, and index VTE phenotype; and 3) fully adjusted models additionally including initial and long-term anticoagulant therapy. All 3 primary models were estimated in the matched cohort. Thus, “fully adjusted” refers to the additional treatment covariates and does not denote a separate matched population. Because treatment variables could represent intermediates or confounders, the fully adjusted estimates were interpreted as treatment-adjusted associations rather than direct or causal effects.

Sensitivity analyses were performed in the full unmatched cohort. Exploratory unadjusted subgroup analyses were conducted among patients without active cancer, patients presenting with PE, patients treated with DOACs, patients younger than 65 years, and patients enrolled in Europe or the Americas. All tests were 2-sided, with a significance threshold of *P* < .05. Subgroup and sensitivity analyses were considered exploratory.

## Results

### Baseline characteristics

The matched cohort included 1792 Hispanic and 5376 non-Hispanic patients with acute VTE. The mean age was 57 ± 18 years, 59% were women, and 11% had active cancer in both groups.

Baseline characteristics were well balanced for matching variables. However, residual imbalances, defined as standardized mean differences >0.10, remained for body weight, chronic lung disease, known thrombophilia, recent surgery, initial fondaparinux use, statin use, antipsychotic use, long-term low-molecular-weight heparin (LMWH) use, long-term DOAC use, and geographic region. For long-term therapy, Hispanic patients were more frequently treated with DOACs (50% vs 39%; standardized mean difference, 0.22) and less frequently treated with LMWH (23% vs 30%; standardized mean difference, 0.16). Geographic distribution differed substantially between groups. Among Hispanic patients, 832 (46%) were enrolled in Europe and 960 (54%) in the Americas; none were enrolled in Asia or Africa. Among non-Hispanic patients, 4736 (88%) were enrolled in Europe, 82 (1.5) in the Americas, 553 (10%) in Asia, and 5 (0.1%) in Africa (Table 1).

**Table 1:** Baseline characteristics of patients with acute VTE by Hispanic vs non-Hispanic status (matched cohort).

Characteristics	Hispanic (*n* = 1792)	Non-Hispanic *(n* = 5376)	Standardized mean difference
Demographic characteristic			
Age, y	57 ± 18	57 ± 18	0.01
Female sex, n (%)	1056 (59)	3168 (59)	0.00
Weight, kg	76 ± 17	79 ± 1 9	0.18
Clinical characteristics			
Active cancer	204 (11)	612 (11)	0.00
Metastatic cancer	105 (5.9)	360 (6.8)	0.03
Prior VTE	176 (9.8)	676 (13)	0.09
CrCl < 60 mL/min	316 (18)	920 (17)	0.01
Liver disease	18 (1.0)	65 (1.2)	0.02
Heart failure	72 (4.1)	195 (3.8)	0.02
Coronary artery disease	75 (4.3)	209 (4.1)	0.01
Diabetes mellitus	194 (11)	673 (13)	0.06
Prior stroke	52 (3.0)	213 (4.2)	0.06
Chronic lung disease	101 (5.6)	446 (8.3)	0.11
Thrombocytopenia	32 (1.8)	96 (1.8)	0.00
Anemia	552 (31)	1730 (32)	0.03
Known thrombophilia	82 (4.6)	386 (7.2)	0.11
Recent surgery	283 (16)	555 (10)	0.16
Recent immobilization	369 (21)	1162 (22)	0.03
Pregnancy or hormone-associated VTE	220 (12)	715 (13)	0.03
Geographic region			
Europe	832 (46)	4736 (88)	0.99
Americas	960 (54)	82 (1.5)	1.43
Asia	0 (0)	553 (10)	0.48
Africa	0 (0)	5 (0.1)	0.04
Index event characteristics			
Pulmonary embolism	1125 (63)	3375 (63)	0.00
Isolated lower-limb DVT	667 (37)	2001 (37)	0.00
Proximal lower-limb DVT	466 (26)	1496 (28)	0.04
Initial anticoagulation therapy			
LMWH	1,515 (85)	4448 (83)	0.05
DOAC	191 (11)	518 (9.6)	0.03
UFH	49 (2.7)	232 (4.3)	0.09
Fondaparinux	9 (0.50)	96 (1.8)	0.12
Long-term anticoagulation therapy			
LMWH	411 (23)	1612 (30)	0.16
DOAC	897 (50)	2108 (39)	0.22
Vitamin K antagonist	436 (24)	1519 (28)	0.09
Other medications and procedures			
Systemic thrombolysis	33 (1.8)	136 (2.5)	0.05
Catheter-directed therapy	20 (1.1)	108 (2.0)	0.07
Pulmonary embolectomy	4 (0.2)	20 (0.4)	0.03
ECMO	5 (0.3)	13 (0.2)	0.01
Inferior vena cava filter	44 (2.5)	86 (1.6)	0.06
Antiplatelets	48 (3.0)	151 (3.2)	0.01
Statins	253 (15)	1025 (21)	0.16
Corticosteroids	164 (10)	412 (8.7)	0.05
Antipsychotics	216 (13)	1057 (22)	0.23

Continuous variables are presented as mean ± SD, and categorical variables as number (percentage). Standardized mean differences >0.10 indicate meaningful imbalance. Geographic region was not included in the matching algorithm and therefore remains imbalanced between groups; this variable was adjusted for in multivariable analyses. CrCl, creatinine clearance; DOAC, direct oral anticoagulant; DVT, deep vein thrombosis; ECMO, extracorporeal membrane oxygenation; LMWH, low-molecular-weight heparin; UFH, unfractionated heparin; VTE, venous thromboembolism.

### Ninety-Day outcomes (matched cohort)

At 90 days, absolute event rates were low in both groups. Recurrent VTE occurred in 1.6% of Hispanic patients and 1.2% of non-Hispanic patients (absolute difference, +0.4 percentage points), whereas major bleeding occurred in 2.1% and 1.7%, respectively (absolute difference, +0.4 percentage points). The composite outcome of recurrent VTE or major bleeding occurred in 3.4% and 2.9%, respectively (absolute difference, +0.5 percentage points). All-cause mortality occurred in 4.1% and 3.9%, respectively (absolute difference, +0.2 percentage points; Table 2). Absolute differences were small across outcomes (Figure 1).

**Table 2:** Ninety-day outcomes after index venous thromboembolism (VTE) by Hispanic vs Non-Hispanic status (matched cohort).

Outcome	Hispanic (*n* = 1792)	Non-Hispanic (*n* = 5376)	absolute difference, percentage points	Standardized mean difference
Recurrent VTE	28 (1.6)	65 (1.2)	+0.4	0.03
Major bleeding	37 (2.1)	93 (1.7)	+0.4	0.02
All-cause mortality	74 (4.1)	212 (3.9)	+0.2	0.01
VTE-related mortality	4 (0.2)	17 (0.3)	–0.1	0.02
Bleeding-related mortality	2 (0.1)	10 (0.2)	–0.1	0.02
Recurrent VTE or major bleeding	61 (3.4)	155 (2.9)	+0.5	0.03

Values are presented as number (percentage). Absolute differences are calculated as Hispanic minus non-Hispanic event rates. Standardized mean differences >0.10 indicate meaningful imbalance.

**Figure 1:**
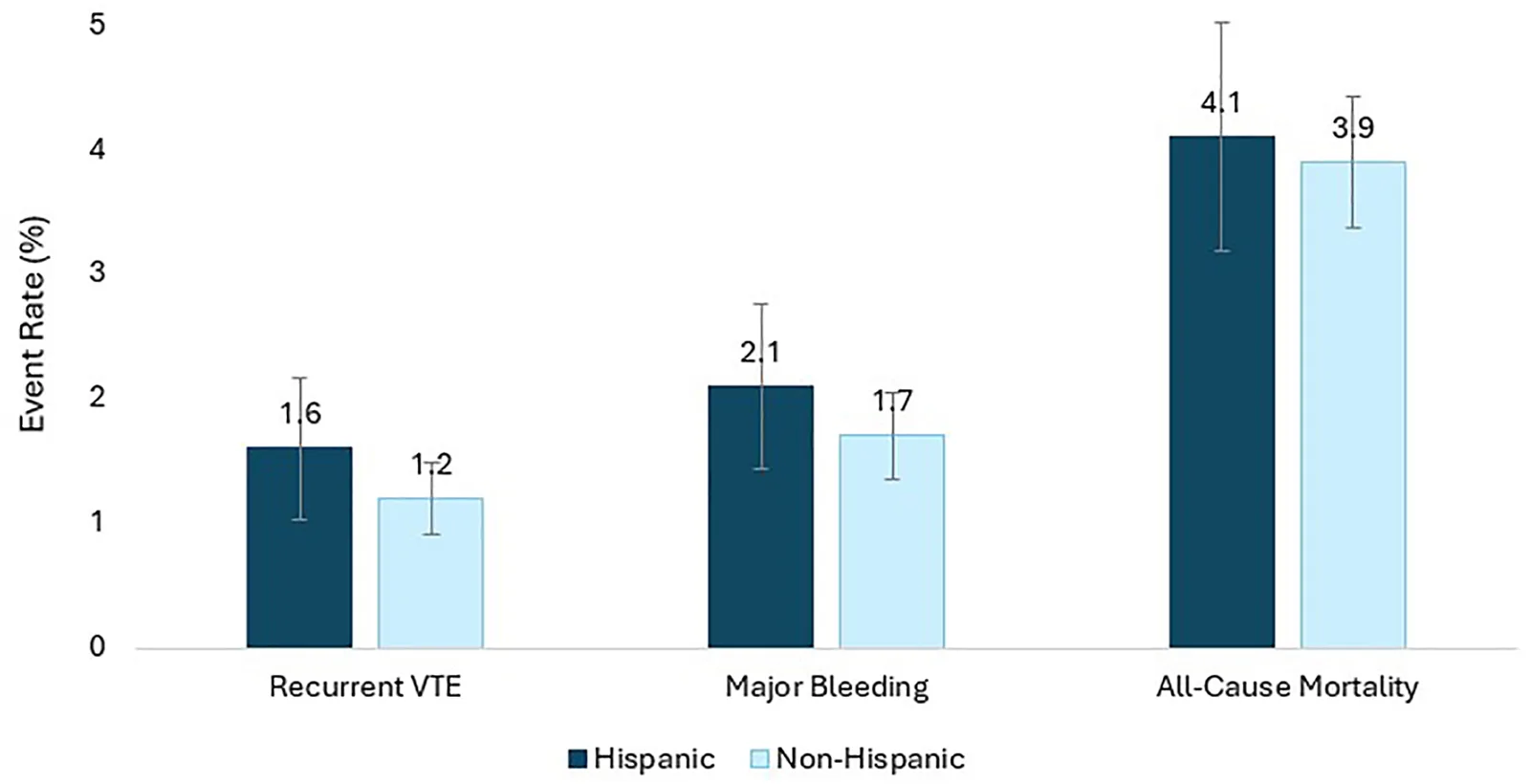
Absolute 90-day event rates by Hispanic Ethnicity in the matched cohort. Bar chart showing absolute rates of recurrent venous thromboembolism (VTE), major bleeding, and all-cause mortality in Hispanic and non-Hispanic patients. Error bars represent 95% CIs. Absolute between-group differences were 0.4 percentage points for recurrent VTE, 0.4 percentage points for major bleeding, and 0.2 percentage points for all-cause mortality.

### Association between Hispanic ethnicity and outcomes

Despite similar crude event rates, adjusted analyses demonstrated differences in relative estimates. In unadjusted analyses, Hispanic ethnicity was not significantly associated with recurrent VTE, major bleeding, or mortality. After clinical adjustment, Hispanic ethnicity was associated with higher odds of recurrent VTE and major bleeding. These associations remained consistent in fully adjusted models: recurrent VTE (OR, 1.90; 95% CI, 1.10-3.28), major bleeding (OR, 1.73; 95% CI, 1.09-2.75), and the composite outcome (OR, 1.77; 95% CI, 1.23-2.54). There was no significant association with all-cause mortality (OR, 1.05; 95% CI, 0.68-1.64) or cause-specific mortality (Table 3, Figure 2).

**Table 3:** Association between Hispanic ethnicity and 90-day outcomes in the matched cohort.

Outcome	Unadjusted OR (95% CI)	Clinically adjusted OR (95% CI)	Fully adjusted OR (95% CI)
Recurrent VTE	1.30 (0.83-2.03)	2.09 (1.21-3.61)^a^	1.90 (1.10-3.28)^b^
Major bleeding	1.20 (0.82-1.76)	1.84 (1.17-2.89)^a^	1.73 (1.09-2.75)^b^
All-cause mortality	1.05 (0.80-1.37)	1.20 (0.81-1.80)	1.05 (0.68-1.64)
VTE-related mortality	0.71 (0.24-2.10)	0.52 (0.11-2.52)	0.52 (0.11- 2.47)
Bleeding-related mortality	0.60 (0.13-2.74)	0.97 (0.14-6.95)	0.75 (0.10-5.55)
Recurrent VTE or major bleeding	1.19 (0.88-1.60)	1.90 (1.33-2.71)^c^	1.77 (1.23-2.54)^a^

Odds ratios (ORs) and 95% CIs were estimated using logistic regression models. Unadjusted models included Hispanic ethnicity and stratification by matched sets. Clinically adjusted models included active cancer; provoking factors (recent surgery, recent immobilization, pregnancy, and hormone-associated VTE); prior VTE; creatinine clearance < 60 mL/min; liver disease; heart failure; chronic lung disease; thrombocytopenia; anemia; geographic region; and index VTE phenotype. Fully adjusted models additionally included initial and long-term anticoagulant therapy. All models were estimated in the matched cohort. VTE, venous thrmboembolism.

a *P* < .01.

b *P*< 0.05.

c *P* < .001.

**Figure 2:**
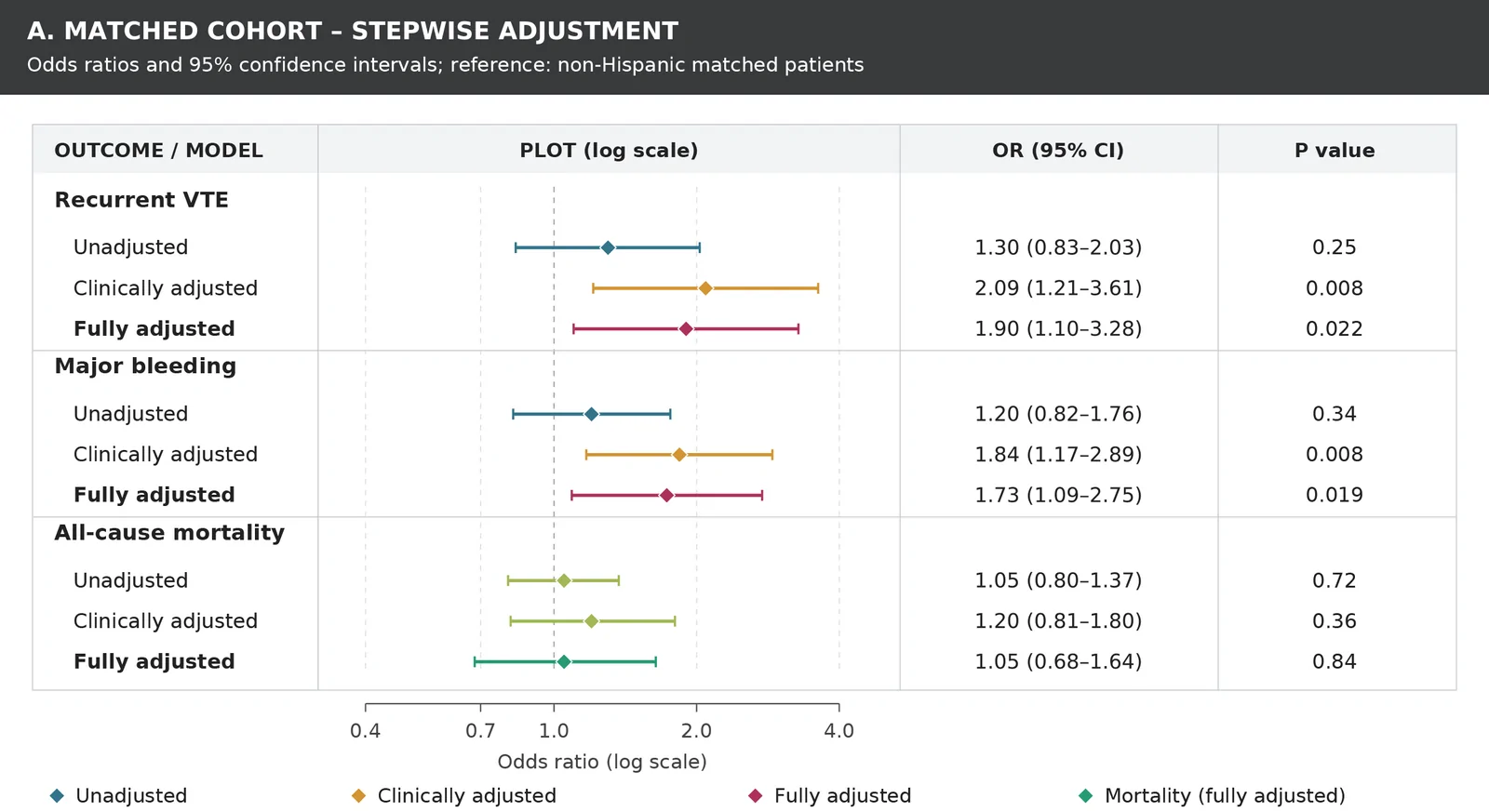
Fully adjusted associations between Hispanic ethnicity and 90-day outcomes in the matched cohort. Forest plot of fully adjusted odds ratios (ORs) and 95% CIs comparing 90-day outcomes among Hispanic vs non-Hispanic patients in the matched cohort. Models included stratification by matched set and adjustment for active cancer; provoking factors, including recent surgery, recent immobilization, pregnancy, and hormone-associated venous thromboembolism (VTE); prior VTE; creatinine clearance <60 mL/min; liver disease; heart failure; chronic lung disease; thrombocytopenia; anemia; geographic region; index VTE phenotype; and initial and long-term anticoagulant therapy. The vertical line indicates the null (OR, 1.0).

### Unmatched cohort analyses

In the full cohort (1,794 Hispanic and 47,483 non-Hispanic patients), crude rates of recurrent VTE, major bleeding, and the composite outcome were similar between groups. However, all-cause mortality was lower among Hispanic patients (4.1% vs 7.0%).

After adjustment for clinical characteristics and treatment variables, Hispanic ethnicity was associated with higher odds of major bleeding (OR, 1.53; 95% CI, 1.04-2.23) and the composite outcome (OR, 1.41; 95% CI, 1.04-1.92), with no significant differences in recurrent VTE or mortality (Figure 3).

**Figure 3:**
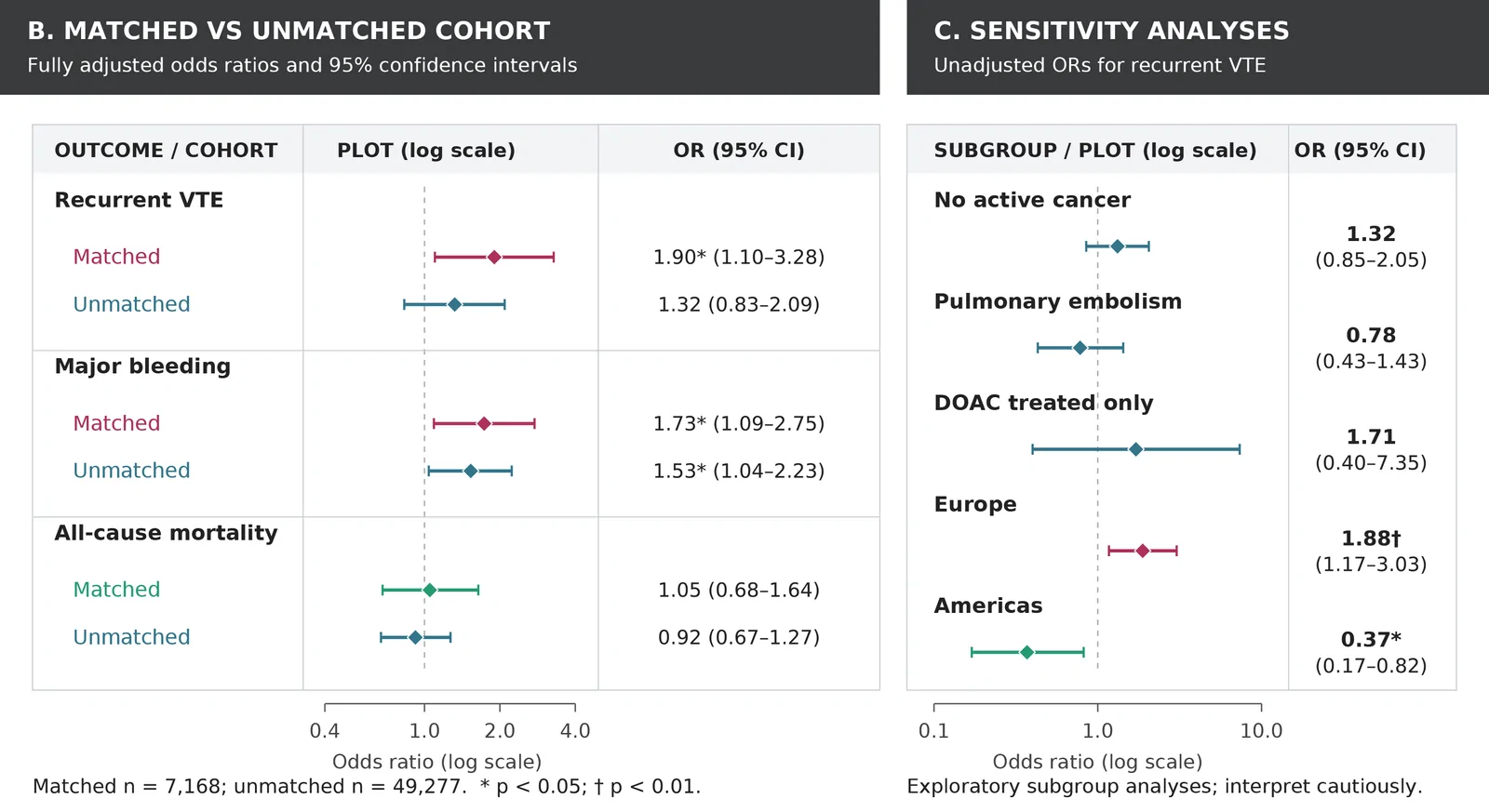
Primary and sensitivity analyses of 90-day outcomes. The left panel presents the fully adjusted odds ratios (ORs) with 95% CIs for recurrent venous thromboembolism (VTE), major bleeding, and all-cause mortality in matched and unmatched cohorts. The right panel presents unadjusted exploratory estimates for recurrent VTE among patients without active cancer, patients presenting with pulmonary embolism (PE), patients treated with direct oral anticoagulants (DOACs), patients younger than 65 years, and patients enrolled in Europe or the Americas. Because the sensitivity analyses were unadjusted, they are not directly comparable with the fully adjusted primary estimates. The vertical line represents the null value (OR = 1.0).

### Sensitivity analyses

In the exploratory unadjusted subgroup analyses, the estimates varied and were generally imprecise. For recurrent VTE, the OR was 1.32 (95% CI, 0.85-2.05) among patients without active cancer, 0.78 (95% CI, 0.43-1.43) among patients presenting with PE, 1.71 (95% CI, 0.40-7.35) among patients treated with DOACs, and 1.12 (95% CI, 0.73-1.74) among patients younger than 65 years. The corresponding major bleeding estimates ranged from 0.90 to 1.13, with all 95% CIs crossing the null. Because these subgroup models were unadjusted, they are not directly comparable with the fully adjusted primary analysis (Supplementary Table).

Exploratory unadjusted analyses stratified by geographic region produced different point estimates in Europe and the Americas. Among patients enrolled in Europe, the ORs comparing Hispanic with non-Hispanic patients were 1.88 (95% CI, 1.17-3.03) for recurrent VTE, 1.46 (95% CI, 0.98-2.17) for major bleeding, and 0.59 (95% CI, 0.41-0.85) for all-cause mortality. Among patients enrolled in the Americas, the corresponding ORs were 0.37 (95% CI, 0.17-0.82), 0.64 (95% CI, 0.28-1.49), and 0.80 (95% CI, 0.51-1.27), respectively. Because these analyses were unadjusted and exploratory, the differing point estimates should not be interpreted as demonstrating that geographic region caused or modified the observed associations.

## 4 Discussion

Hispanic ethnicity was associated with higher adjusted odds of recurrent VTE and major bleeding during the first 90 days after diagnosis, but not with mortality. Absolute risk differences were ≤0.5 percentage points across outcomes, indicating that the clinical magnitude of these differences is modest. This distinction is clinically important, as relative differences may overstate the perceived magnitude of risk when the absolute event rates are low. Because the registry did not directly measure socioeconomic conditions, healthcare access, structural inequities, or cultural factors, the observed associations cannot be attributed to any of these mechanisms. Hispanic ethnicity should be interpreted as a broad, self-identified sociocultural classification rather than a discrete biological exposure. Potential explanations for the observed associations include residual confounding by patient characteristics, treatment selection, differences among participating centers, geographic composition, and unmeasured socioeconomic or healthcare-related factors. The present analysis cannot distinguish among these explanations or exclude contributions from other clinical or biological characteristics.

The early postdiagnosis period represents a vulnerable phase characterized by dynamic changes in anticoagulant therapy, transitions of care, and evolving clinical stability [18,19]. Several explanations could account for the adjusted associations, including residual confounding by patient characteristics, imbalances that persisted after matching, differences in treatment selection, differences among participating centers, geographic composition, and unmeasured socioeconomic or health care-related factors. Because many of these constructs were not directly measured, the study cannot determine their relative contributions or establish a causal mechanism. The absence of an adjusted association with mortality indicates only that mortality did not differ between groups; it should not be interpreted as evidence supporting a particular explanation. Because treatment variables could represent intermediates or confounders, the fully adjusted estimates were interpreted as treatment-adjusted associations rather than direct or causal effects.

Differences in anticoagulant selection represent 1 potential explanation for the findings. Hispanic patients were more frequently treated with DOACs and less frequently with LMWH; however, the registry data did not establish why these treatments were selected or whether treatment differences mediated the observed associations. Other potential explanations include differences in treatment continuity, follow-up, and patient-level barriers such as health literacy, which may influence recurrence and bleeding risk [20,21]. Because these variables were not directly captured, they should be considered hypotheses for future study rather than explanations established by the present analysis. Given the small absolute differences and observational design, these findings do not support using ethnicity to guide anticoagulation management or attributing the observed association to a specific care process. Future studies incorporating direct measures of these factors are needed to determine whether strategies such as a standardized follow-up and structured anticoagulation transitions improve outcomes across patient populations.

Exploratory unadjusted subgroup analyses produced lower Hispanic-vs-non-Hispanic estimates in the Americas than in Europe, particularly recurrent VTE. These findings should be interpreted cautiously because the geographic distribution of participants was uneven, the subgroup analyses were unadjusted, and differences between regions could reflect patient characteristics, participating centers, treatment patterns, other unmeasured factors, or chance. Because of limited geographic overlap between exposure groups, these exploratory stratified estimates should not be interpreted as evidence of effect modification.

Prior multinational studies have reported geographic variation in VTE presentation, management, and outcomes [3,11,22,23]. These studies support the plausibility of regional variation but do not establish which patient-, center-, treatment-, or healthcare-system-level factors account for the differences. Similarly, the present analysis cannot determine whether the observed regional variation reflects healthcare-system characteristics or other measured or unmeasured factors.

This study has several limitations. First, as an observational registry analysis, it cannot establish causality. Matching balanced only the measured variables included in the matching procedures and could not account for unmeasured confounding. Residual differences remained in characteristics associated with VTE outcomes, including body weight, known thrombophilia, and anticoagulant selection; therefore, residual confounding may affect the adjusted estimates, particularly given the small absolute differences between groups. Geographic overlap between the exposure groups was limited: all Hispanic participants were enrolled in Europe or the Americas, whereas the non-Hispanic group also included patients enrolled in Asia and Africa. In addition, the proportions enrolled in Europe and the Americas differed substantially between groups. Although geographic region was included in the adjusted models, this imbalance may contribute to residual confounding and limit interpretation of the region-stratified analyses. Second, the registry did not capture several potentially important determinants of outcomes, including socioeconomic factors, healthcare access, treatment adherence, transitions of care, and center-specific practice patterns.

In summary, self-identified Hispanic ethnicity was associated with modestly higher adjusted odds of early recurrent VTE and major bleeding, but not mortality. Absolute differences in event rates were small. Exploratory subgroup analyses produced lower Hispanic vs non-Hispanic estimates among patients enrolled in the Americas than among those enrolled in Europe; however, these findings do not establish that geography or healthcare-system factors caused the observed differences. Residual confounding by patient-, treatment-, and region-level factors remain possible. Future studies incorporating direct measures of healthcare access, socioeconomic conditions, treatment adherence, care delivery, and center-level practices are needed to clarify the mechanisms underlying these associations.
