# Supplementary material for: Early outcomes after venous thromboembolism in Hispanic and non-Hispanic patients: insights from the Registro Informatizado de Enfermedad TromboEmbólica Registry
Source: Res Pract Thromb Haemost. 2026 Sep 15;10(7):106969. doi: 10.1016/j.rpth.2026.106969 (PMC13675914; doi:10.1016/j.rpth.2026.106969)
Supplement: Supplementary file 1 [file mmc1.docx]

**Supplementary Table. Unadjusted Exploratory Subgroup Analyses of 90-Day Outcomes**

| **Model / restriction** | **Recurrent VTE**  **unadjusted OR**  **(95% CI)** | **Major bleeding**  **unadjusted OR**  **(95% CI)** | **All-cause mortality**  **unadjusted OR**  **(95% CI)** |
| --- | --- | --- | --- |
| No active cancer | 1.32 (0.85-2.05) | 0.94 (0.63-1.39) | 0.48 (0.33-0.70) ^‡^ |
| Pulmonary embolism only | 0.78 (0.43-1.43) | 0.90 (0.60-1.34) | 0.50 (0.37-0.67) ^‡^ |
| DOAC only | 1.71 (0.40-7.35) | 1.12 (0.15-8.47) | 1.90 (0.67-5.38) |
| Age < 65 years | 1.12 (0.73-1.74) | 1.13 (0.70-1.83) | 0.68 (0.49-0.96) ^*^ |
| Europe | 1.88 (1.17-3.03) ^†^ | 1.46 (0.98-2.17) | 0.59 (0.41-0.85) ^†^ |
| Americas | 0.37 (0.17-0.82) ^*^ | 0.64 (0.28-1.49) | 0.80 (0.51-1.27) |

Odds ratios (ORs) and 95% confidence intervals (CIs) are from unadjusted models. Analyses are exploratory and should be interpreted cautiously.

**Principal Investigator:** Manuel Monreal.

**RIETE Steering Committee Members:** Paolo Prandoni, Benjamin Brenner and Dominique Farge-Bancel.

**RIETE National Coordinators:** Raquel Barba (Spain), Peter Verhamme (Belgium), Hugo Hyung Bok Yoo (Brazil), Radovan Malý (Czech Republic), Laurent Bertoletti (France), Sebastian Schellong (Germany), Inna Tzoran (Israel), Pierpaolo Di Micco (Italy), Abilio Reis (Portugal), Marijan Bosevski (R. North Macedonia), Lucia Mazzolai (Switzerland), Joseph A. Caprini (USA), My Hanh Bui (Vietnam).

**RIETE Registry Coordinating Center:** S & H Medical Science Service.

**APPENDIX**

Members of the RIETE Group

SPAIN**: Abad-Fernández A, Afonso-Nuez L, Agudo P, Aibar J, Alberich-Conesa A, Álvarez-Albarrán J, Amado C, Angelina-García M, Arcelus JI, Ballaz A, Barba R, Barrón M, Barrón-Andrés B,** **Bascuñana J, Beddar-Chaib F, Blanco-Molina Á, Cabello-Rojano I, Chamorro N, Chasco L, Claver G, Creu-Paris J, Criado J, De Juana-Izquierdo C, Del Toro J, Demelo-Rodríguez P, Díaz-Anichiarico JD, Díaz-Pedroche MC, Díaz-Peromingo JA, Durán D, Esquerdo-Serrano P, Falgá C, Fernández-Martínez de Septién C, Ferreiro-Celeiro J, Fidalgo Á, Formica A, Francisco I, Gabara C, Galeano-Valle F, García-Bragado F, García-Ortega A, Gavín-Sebastián O, Gil-Díaz A, Girona E, Gómez-Cuervo C,** **González-Olmedo J,** **Gorostidi-Álvarez I,** **Gorostidi-Pérez J,** **Guirado L, Gutiérrez-Guisado J, Hernández-Blasco L,** **Hernández-Borge J, Jiménez D, Jiménez R, Jou I, Joya MD, Larrauri A, Llamas P, López-Miguel P, López-Núñez JJ, López-Ruiz A, López-Sáez JB, Lorenzo A, Lucas-García P, Madridano O, Marchena PJ, Martín-del Pozo M, Monreal M, Monzón-Escribano L, Moreno-Fernández A, Nieto JA, Núñez-Fernández MJ, Olid M, Ordieres-Ortega L, Ortiz M, Otálora S, Pagán J, Parra-Caballero P, Pedrajas JM, Pérez-Pinar M, Pérez-Ductor C, Peris ML, Pesce ML, Puche G, Rodríguez-Cobo A, Rojas-Piñero MD, Ruiz-Artacho P, Ruiz-Giménez N, Sánchez-Camacho M, Sancho T, Sendín V, Sidawi-Urbano T, Sierra-Palomares G, Sigüenza P, Sindín-Martín  L, Solé A, Teres-Pueyo L, Toda MR, Trujillo-Santos J, Tudela L, Valle R, Varona JF, Vega-Romero E, Villares P,** AUSTRIA**: Ay C, Nopp S, Pabinger I,** BELGIUM**: Erard M, Van Thillo Q, Verhamme P,** BRAZIL**:** **Yoo HHB,** COLOMBIA**: Carbonell SE, Gómez-Mesa JE, Montenegro AC, Roa J,** CZECH REPUBLIC**: Grenar P, Hirmerova J, Malý R,** FRANCE**: Accassat S, Benarroch S, Bertoletti L, Bura-Riviere A, Catella J, Chopard R, Duong R, Espitia O, Leclercq B, Mahé I, Millet G, Moustafa F, Poenou G, Sarlon-Bartoli G,** GERMANY**: Schellong S,** IRAN**: Jenab Y, Khodayari A, Sadeghipour P, Yadangi S,** ISRAEL**: Brenner B, Dally N, Tzoran I,** ITALY**: Barillari G, Bilora F, Bortoluzzi C, Bortoluzzi M, Brandolin B, Buso G, Ciammaichella MM, Di Micco P, Guida A, Imbalzano E, Licci A, Marcon C, Mastroiacovo D, Mazzarelli U, Pesavento R,** **Pizzuti V, Poz A, Prandoni P, Provisione M, Scarinzi P, Visonà A, Vo Hong N, Zalunardo B,** LATVIA**:** **Cesnieks H,** **Skride A,** **Zicans M,** PORTUGAL**: Fonseca S, Martins MD, Pereira-Fontes C,** REPUBLIC OF NORTH MACEDONIA**: Bosevski M,** **Boshevska M,** SWITZERLAND**: Barco S, Mazzolai L,** USA**: Angiolillo DJ, Bikdeli B, Caprini JA, Ortega-Paz L, Tafur AJ,** VIETNAM**: Bui MH, Nguyen ST, Tran GB.**
